# Mechanism of Action of Acetazolamide and Idiopathic Intracranial Hypertension

**DOI:** 10.3389/fneur.2015.00013

**Published:** 2015-02-03

**Authors:** J. Gordon Millichap, John J. Millichap

**Affiliations:** ^1^Department of Pediatrics, Northwestern University Feinberg School of Medicine, Chicago, IL, USA; ^2^Department of Neurology, Northwestern University Feinberg School of Medicine, Chicago, IL, USA; ^3^Division of Neurology, Ann & Robert H. Lurie Children’s Hospital of Chicago, Chicago, IL, USA

**Keywords:** carbonic anhydrase, acetazolamide, idiopathic intracranial hypertension, pharmacology, papilledema

We wish to comment on the correspondence regarding the question of efficacy and mechanism of action of acetazolamide in the treatment of idiopathic intracranial hypertension (IIH), published in JAMA September 10, 2014 (1). Whereas Sinclair et al. (2) minimize the benefits and favor a theory of weight loss as the reason for a small improvement in symptoms and reduced papilledema, Wall et al. (3) hold that acetazolamide has a direct effect on papilledema and intracranial pressure, and significantly improves visual field function in a patient with IIH. The distribution of carbonic anhydrase and acetazolamide in the brain and how that relates to the mechanism of action of acetazolamide in IIH are not addressed.

In the 1950s, a similar discussion concerned the mechanism of acetazolamide in the treatment of epileptic seizures. Initially, it was thought that the effect was indirect via the kidneys and was related to an induced metabolic acidosis. Subsequently, acetazolamide was shown to prevent seizures in nephrectomized animals; the anticonvulsant effect was independent of the effect on the kidneys and was caused by a direct inhibition of carbonic anhydrase in the brain (4).

In patients with IIH treated with acetazolamide, the inhibition of the enzyme in the choroid plexus results in a reduction of CSF production and flow. The acid–base status of the patient may also alter the distribution of acetazolamide in the CSF and brain, but its effect on the CSF flow is secondary to that mediated by the choroid plexus. Based on the pharmacology and distribution of acetazolamide and carbonic anhydrase in the brain, the theory that emphasizes the effect of acetazolamide on CSF production in IIH is most likely primary and direct, and weight loss, when recognized as a factor, is secondary and indirect, and frequently the result of toxic doses in excess of the amount needed for complete enzyme inhibition.

## Conflict of Interest Statement

The authors declare that the research was conducted in the absence of any commercial or financial relationships that could be construed as a potential conflict of interest.
